# Perinatal HIV-1 transmission: Fc gamma receptor variability associates with maternal infectiousness and infant susceptibility

**DOI:** 10.1186/s12977-016-0272-y

**Published:** 2016-06-10

**Authors:** Ria Lassaunière, Alfred Musekiwa, Glenda E. Gray, Louise Kuhn, Caroline T. Tiemessen

**Affiliations:** Centre for HIV and STIs, National Institute for Communicable Diseases (NHLS), Johannesburg, South Africa; Faculty of Health Sciences, University of the Witwatersrand, Johannesburg, South Africa; International Emerging Infections Programme, South Africa Global Disease Detection Centre, Centers for Disease Control and Prevention (CDC), Pretoria, South Africa; Perinatal HIV Research Unit, Chris Hani Baragwanath Hospital, Soweto, South Africa; Gertrude H. Sergievsky Centre, College of Physicians and Surgeons, Columbia University, New York, NY USA; Department of Epidemiology, Mailman School of Public Health, Columbia University, New York, NY USA

**Keywords:** HIV-1, Vertical infectious disease transmission, Risk factors, IgG receptors, Alleles, Antibody-dependent cell cytotoxicity, Phagocytosis

## Abstract

**Background:**

Accumulating data suggest that immune effector functions mediated through the Fc portion of HIV-1-specific immunoglobulin G (IgG) are a key component of HIV-1 protective immunity, affecting both disease progression and HIV-1 acquisition. Through studying Fc gamma receptor (FcγR) variants known to alter IgG Fc-mediated immune responses, we indirectly assessed the role of FcγR-mediated effector functions in modulating perinatal HIV-1 transmission risk. In this study, genotypic data from 79 HIV-1 infected mothers and 78 HIV-1 infected infants (transmitting cases) were compared to 234 HIV-1 infected mothers and 235 HIV-1 exposed-uninfected infants (non-transmitting controls). Associations, unadjusted and adjusted for multiple comparisons, were assessed for overall transmission and according to mode of transmission—intrapartum (n = 31), in utero (n = 20), in utero-enriched (n = 48).

**Results:**

The maternal FcγRIIIa-158V allele that confers enhanced antibody binding affinity and antibody-dependent cellular cytotoxicity capacity significantly associated with reduced HIV-1 transmission [odds ratio (OR) 0.47, 95 % confidence interval (CI) 0.28–0.79, P = 0.004; P_Bonf_ > 0.05]. In particular, the FcγRIIIa-158V allele was underrepresented in the in utero transmitting group (P = 0.048; P_Bonf_ > 0.05) and in utero-enriched transmitting groups (P = 0.0001; P_Bonf_ < 0.01). In both mother and infant, possession of an FcγRIIIb-HNA1b allotype that reduces neutrophil-mediated effector functions associated with increased transmission (OR 1.87, 95 % CI 1.08–3.21, P = 0.025; P_Bonf_ > 0.05) and acquisition (OR 1.91, 95 % CI 1.11–3.30, P = 0.020; P_Bonf_ > 0.05), respectively. Conversely, the infant FcγRIIIb-HNA1a|1a genotype was significantly protective of perinatal HIV-1 acquisition (OR 0.42, 95 % CI 0.18–0.96, P = 0.040; P_Bonf_ > 0.05).

**Conclusions:**

The findings of this study suggest a potential role for FcγR-mediated effector functions in perinatal HIV-1 transmission. However, future studies are required to validate the findings of this study, in particular associations that did not retain significance after adjustment for multiple comparisons.

**Electronic supplementary material:**

The online version of this article (doi:10.1186/s12977-016-0272-y) contains supplementary material, which is available to authorized users.

## Background

Beyond neutralization, immunoglobulin G (IgG) has the capacity to recruit potent effector functions of the innate immune system through engagement with Fc gamma receptors (FcγRs), which are widely expressed throughout the haematopoietic system. Directly or indirectly, FcγRs mediate antiviral processes that include antibody-dependent cellular cytotoxicity (ADCC), antibody-dependent cellular phagocytosis (ADCP), respiratory burst, antigen display, antibody production, cell activation, and release of inflammatory mediators [1].

FcγR-mediated effector functions are increasingly recognized as a component of HIV-1 protective immunity [2]. However, the role of these effector functions in modulating perinatal HIV-1 transmission risk is currently undefined. Given the contribution of FcγR-mediated effector functions to eliminating cell-free and cell-associated virus, these processes may modify the infectiousness of an HIV-1 infected mother. In addition, transplacental transferred anti-HIV-1 IgG may recruit innate immune effector functions in the foetus/infant through engaging FcγRs expressed on foetal/infant immune cells, and in this manner modify the infant’s susceptibility to HIV-1 acquisition.

In vivo, FcγR-mediated effector functions are governed by a balance between activating and inhibitory FcγRs [3]. This balance is perturbed by functionally significant genotypic variants that modulate cellular activation and ultimately effector function capability. These include gene duplication/deletion that affects FcγR surface density [4, 5] and amino acid changes that alter the receptor’s binding affinity for antibody subclasses (FcγRIIa-H131R and FcγRIIIa-F158V) [6, 7], subcellular localization (FcγRIIb-I232T) [8], glycosylation patterns (FcγRIIIb-HNA1a|b|c) [9, 10], and the expression of a functional molecule (FcγRIIc-X57Q and c.798+1A>G) [11, 12].

Using these variants as a proxy for functional capability, this study indirectly assessed the potential role of FcγR-mediated effector functions in mother-to-child transmission of HIV-1. Due to the exploratory nature of the study, associations are reported unadjusted for multiple comparisons. However, adjusted associations were also considered. Our findings highlight a potential role for the FcγRIIIa-F158V variant in modulating maternal infectiousness, while in both mother and infant the FcγRIIIb-HNA1a|b|c variant associated with HIV-1 transmission.

## Results

### Cohort

A nested case–control study was undertaken to investigate *FCGR* variability in HIV-1 infected mothers and their infants recruited as part of four perinatal cohorts at two hospitals in Johannesburg, South Africa [13]. Overall, the four cohorts comprised 849 HIV-1 infected mothers and their infants, of whom 83 (10 %) acquired HIV-1 perinatally. In the present study, *FCGR* genotypic data from 79 HIV-1 infected mothers and 78 HIV-1 infected infants (transmitting cases) were compared with 234 HIV-1 infected mothers and 235 uninfected infants (non-transmitting controls). Mode of transmission was defined according to the presence/absence of detectable HIV-1 DNA in the infant at birth and 6 weeks of age. Infants that tested HIV-1 positive at 6 weeks of age, but who were negative at birth, were considered to be infected intrapartum (during labour and delivery), while infants that tested HIV-1 positive at birth were considered infected in utero. Infants that were HIV-1 positive at 6 weeks, but had no birth sample, were categorized as ‘undetermined’. Since 25/28 (89.2 %) mothers in the ‘undetermined’ category received drug interventions known to reduce intrapartum transmission [14–16], it was concluded that the majority of infants in this group were likely infected in utero and was thus combined with the in utero group to form an in utero-enriched group.

Transmitting mothers had significantly higher HIV-1 plasma viral loads and lower CD4^+^ T cell counts compared to non-transmitting mothers (Table 1). In addition, infants infected in utero had a significantly lower mean birth weight compared to exposed-uninfected infants. Maternal age, parity, mode of delivery, gestation, child sex, and reported breast feeding did not differ significantly between transmitting mothers (total, intrapartum or in utero) and non-transmitting mothers.

**Table 1:** Demographic and clinical characteristics of mothers and infants

Maternal viral load (log_10_ copies/ml)	Non-transmitting (*N* = 234)^a^		Total transmitting (*N* = 79)		Intrapartum transmitting (*N* = 31)		In utero transmitting (*N* = 20)^b^		In utero-enriched transmitting (*N* = 48)	
	N^c^		N^c^		N^c^		N^c^		N^c^	
Median (IQR)	218	4.08 (3.20–4.67)	71	4.77 (3.77–5.34)***	27	4.77 (3.77–5.26)**	18	4.89 (4.20–5.47)***	44	4.81 (3.78–5.44)***
Maternal CD4^+^ T cell count										
Mean (std)	217	520 (275)	70	418 (222)**	27	402 (179)*	15	409 (276)	43	428 (247)*
Maternal age (years)										
Mean (std)	232	26.9 (5.1)	78	27.6 (5.2)	30	26.7 (5.0)	20	27.5 (5.5)	48	28.2 (5.2)
Parity										
Mean (std)	231	2.1 (1.0)	77	2.3 (1.2)	29	2.3 (1.2)	20	2.2 (1.2)	48	2.3 (1.2)
Mode of delivery [N (%)]										
Caesarean section	232	17 (7.3)	77	10 (13.0)	29	2 (6.9)	20	3 (15.0)	48	8 (16.7)
Gestation [*N* (%)]										
Preterm <37 weeks	215	27 (12.6)	70	12 (17.1)	25	7 (28.0)	19	4 (21.1)	45	5 (11.1)
Child sex [*N* (%)]										
Male	234	101 (43.1)	79	39 (49.4)	31	18 (58.0)	20	8 (40.0)	48	21 (43.8)
Birth weight (g)										
Mean (std)	231	2980 (453)	78	2889 (442)	30	2943 (400)	20	2784 (320)*	48	2856 (468)
Breast fed *N* (%)										
>3 days	233	34 (14.6)	78	10 (12.8)	30	5 (16.7)	20	2 (10.0)	48	5 (10.4)
Antiretrovirals										
Nevirapine	234	114 (48.7)	79	47 (59.5)	31	11 (35.5)	20	13 (65.0)	48	36 (75.0)**
Triple drug therapy	234	6 (2.6)	79	2 (2.5)	31	0	20	0	48	2 (4.2)
Other drugs^d^	234	11 (4.7)	79	6 (7.6)	31	3 (9.7)	20	1 (5.0)	48	3 (6.3)

For comparisons with non-transmitting mothers: * P < 0.05; ** P < 0.01; *** P < 0.001

^a^Five unmatched mothers

^b^One unmatched mother

^c^Number of participants for whom data were available

^d^Different regimens of zidovudine (AZT) and lamivudine (3TC)

### Variants not detected in the study cohort

The FcγRIIb 2B.4 promoter haplotype (c.-386C/c.-120A) and expression of functional FcγRIIc are rare to absent in Black South African individuals [17]. Accordingly, in the present cohort of Black South African mothers and infants, none possessed the FcγRIIb 2B.4 promoter haplotype. Furthermore, despite 84/313 (25.3 %) mothers and 81/313 (25.9 %) infants bearing an FcγRIIc-Q57 allele, only one non-transmitting mother expressed functional FcγRIIc as predicted by the *FCGR2C* c.798+1A>G splice-site variant [12].

### *FCGR* copy number variability

**Fig. 1:**
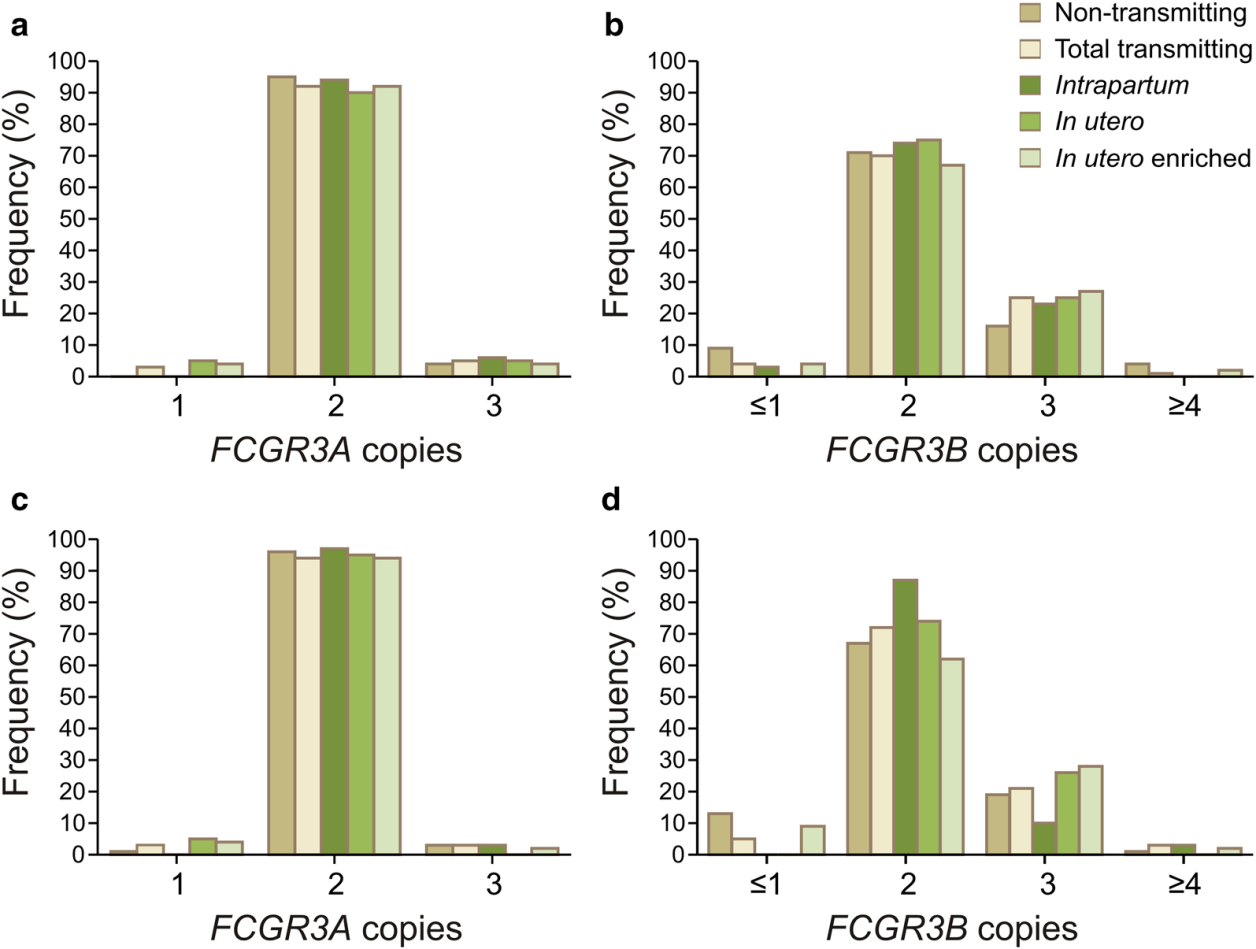
The distribution of *FCGR3A* and *FCGR3B* gene copy number in HIV-1 infected mothers (**a**, **b**, respectively) and their infants (**c**, **d**, respectively)

The frequency of *FCGR3A* gene copy number variability (CNV) was low, occurring in 17/313 (5.4 %) mothers and 14/313 (4.5 %) infants (Fig. 1), and did not associate with perinatal HIV-1 transmission (P > 0.05 for all comparisons; Additional file 1: Table S1). *FCGR3B* gene CNV was observed more frequently in 92/313 (29.4 %) mothers and 100/313 (31.9 %) infants (Fig. 1). The overall distribution of *FCGR3B* gene copy number was significantly different between exposed-uninfected infants and intrapartum infected infants (P = 0.029), with the intrapartum infected group having fewer *FCGR3B* gene duplications and no gene deletions (Additional file 1: Table S1). Maternal *FCGR3B* gene CNV did not associate with HIV-1 transmission (P > 0.05 for all comparisons; Additional file 1: Table S1).

### FcγR variants and infectiousness of the transmitter/mother

To determine if FcγR variants were associated with the infectiousness of the mother, HIV-1 transmission was assessed according to maternal genotypes and allele carriage in a univariate and multivariate model (Table 2, 3, respectively). Overall, the maternal FcγRIIIa-F158V variant significantly associated with HIV-1 transmission (P = 0.017), while a trend was observed for the FcγRIIIb-HNA1a|b|c variant (P = 0.058).

**Table 2:** FcγR genotypes and allele carriage in HIV-1 non-transmitting and transmitting mothers

	Non-transmitting	Total transmitting				Intrapartum transmitting			
	N (%)	N (%)	OR (95 % CI)	P value	P_Bonf_	N (%)	OR (95 % CI)	P value	P_Bonf_
*FcγRIIa (rs1801274)*	Overall association			P = 0.379	ns			P = 0.688	ns
Genotype									
131HH (ref)	60 (25.6)	15 (19.0)	1			6 (19.4)	1		
131HR	106 (45.3)	36 (45.6)	1.36 (0.69–2.68)	P = 0.378	ns	14 (45.2)	1.32 (0.48–3.62)	P = 0.558	ns
131RR	68 (29.1)	28 (35.4)	1.65 (0.80–3.37)	P = 0.172	ns	11 (35.5)	1.62 (0.56–4.64)	P = 0.371	ns
Allele carriage									
≥1 131H allele	166 (70.9)	51 (64.6)	0.75 (0.43–1.28)	P = 0.288	ns	20 (64.5)	0.74 (0.34–1.64)	P = 0.464	ns
≥1 131R allele	174 (74.4)	64 (81.0)	1.47 (0.78–2.77)	P = 0.233	ns	25 (80.6)	1.44 (0.56–3.67)	P = 0.449	ns
*FcγRIIb (rs1050501)*	Overall association			P = 0.194	ns			P = 0.397	ns
Genotype									
232II (ref)	113 (48.3)	32 (40.5)	1			12 (38.7)	1		
232IT	103 (44.0)	36 (45.6)	1.23 (0.71–2.13)	P = 0.450	ns	15 (48.4)	1.37 (0.61–3.07)	P = 0.442	ns
232TT	18 (7.7)	11 (13.9)	2.16 (0.93–5.03)	P = 0.075	ns	4 (12.9)	2.09 (0.61–7.20)	P = 0.242	ns
Allele carriage									
≥1 232I allele	216 (92.3)	68 (86.3)	0.52 (0.23–1.14)	P = 0.103	ns	27 (87.1)	0.56 (0.18–1.79)	P = 0.239	ns
≥1 232T allele	121 (51.7)	47 (59.5)	1.37 (0.82–2.30)	P = 0.231	ns	19 (61.3)	1.48 (0.69–3.18)	P = 0.317	ns
*FcγRIIIa (rs396991)*	Overall association			P = 0.017	ns			P = 0.380	ns
Genotype									
158F/FF/FF (ref)	76 (32.5)	40 (50.6)	1			10 (32.3)	1		
158FV/FFV/FVV	121 (51.7)	31 (39.2)	0.49 (0.28–0.84)	*P* *=* *0.010*	ns	19 (61.3)	1.19 (0.53–2.70)	P = 0.672	ns
158V/VV	36 (15.4)	8 (10.1)	0.41 (0.17–0.97)	*P* *=* *0.041*	ns	2 (6.5)	0.41 (0.09–1.97)	P = 0.266	ns
Allele carriage									
≥1 158F allele	197 (84.2)	71 (89.9)	1.67 (0.74–3.75)	P = 0.217	ns	29 (93.5)	2.72 (0.62–11.91)	P = 0.183	ns
≥1 158V allele	157 (67.1)	39 (49.4)	0.47(0.28–0.79)	*P* *=* *0.004*	ns	21 (67.7)	1.01 (0.45–2.25)	P = 0.980	ns
*FcγRIIIb*	Overall association			P = 0.058	ns			P = 0.647	ns
Genotype									
HNA1a+/1b−/1c−	51 (21.8)	13 (16.5)	0.68 (0.32–1.44)	P = 0.315	ns	4 (12.9)	0.51 (0.15–1.70)	P = 0.276	ns
HNA1a−/1b+/1c−	23 (9.8)	7 (8.9)	0.81 (0.31–2.11)	P = 0.668	ns	4 (12.9)	1.14 (0.33–3.92)	P = 0.837	ns
HNA1a−/1b−/1c+	13 (5.6)	0 (0)	–			0 (0)	–		
HNA1a+/1b+/1c− (ref)	72 (30.8)	27 (34.2)	1			11 (35.5)	1		
HNA1a+/1b−/1c+	40 (17.1)	11 (13.9)	0.73 (0.33–1.63)	P = 0.448	ns	5 (16.1)	0.82 (0.27–2.52)	P = 0.727	ns
HNA1a−/1b+/1c+	22 (9.4)	17 (21.5)	2.06 (0.95–4.46)	P = 0.066	ns	5 (16.1)	1.49 (0.47–4.75)	P = 0.502	ns
HNA1a+/1b+/1c+	12 (5.1)	4 (5.1)	0.89 (0.26–3.00)	P = 0.849	ns	2 (6.5)	1.09 (0.21–5.54)	P = 0.916	ns
Allele carriage									
≥1 HNA1a allotype	175 (74.8)	55 (69.6)	0.77 (0.44–1.36)	P = 0.369	ns	22 (71.0)	0.82 (0.36–1.89)	P = 0.648	ns
≥1 HNA1b allotype	129 (55.1)	55 (69.6)	1.87 (1.08–3.21)	*P* *=* *0.025*	ns	22 (71.0)	1.99 (0.88–4.50)	P = 0.099	ns
≥1 HNA1c allotype	87 (37.2)	32 (40.5)	1.15 (0.68–1.94)	P = 0.599	ns	12 (38.7)	1.07 (0.49–2.30)	P = 0.869	ns

	In utero transmitting				In utero-enriched transmitting			
	N (%)	OR (95 % CI)	P value	P_Bonf_	N (%)	OR (95 % CI)	P value	P_Bonf_
*FcγRIIa (rs1801274)*			P = 0.182	ns			P = 0.545	ns
Genotype								
131HH (ref)	2 (10.0)	1			9 (18.8)	1		
131HR	9 (45.0)	2.55 (0.53–12.17)	P = 0.241	ns	22 (45.8)	1.38 (0.60–3.20)	P = 0.447	ns
131RR	9 (45.0)	3.97 (0.83–19.10)	P = 0.085	ns	17 (35.4)	1.67 (0.69–4.02)	P = 0.225	ns
Allele carriage								
≥1 131H allele	11 (55.0)	0.50 (0.20–1.26)	P = 0.143	ns	31 (64.6)	0.75 (0.39–1.44)	P = 0.383	ns
≥1 131R allele	18 (90.0)	3.10 (0.70–13.77)	P = 0.136	ns	39 (81.3)	1.49 (0.68–3.27)	P = 0.314	ns
*FcγRIIb (rs1050501)*			P = 0.125	ns			P = 0.274	ns
Genotype								
232II (ref)	10 (50.0)	1			20 (41.7)	1		
232IT	6 (30.0)	0.66 (0.23–1.87)	P = 0.434	ns	21 (43.8)	1.15 (0.59–2.25)	P = 0.678	ns
232TT	4 (20.0)	2.51 (0.71–8.87)	P = 0.153	ns	7 (14.6)	2.20 (0.81–5.94)	P = 0.121	ns
Allele carriage								
≥1 232I allele	16 (80.0)	0.33 (0.10–1.10)	P = 0.072	ns	41 (85.4)	0.49 (0.19–1.24)	P = 0.133	ns
≥1 232T allele	10 (50.0)	0.93 (0.37–2.33)	P = 0.883	ns	28 (58.3)	1.31 (0.70–2.45)	P = 0.403	ns
*FcγRIIIa (rs396991)*			P = 0.137	ns			*P* *=* *0.0004*	*0.017*
Genotype								
158F/FF/FF (ref)	11 (55.0)	1			30 (62.5)	1		
158FV/FFV/FVV	8 (40.0)	0.46 (0.18–1.19)	P = 0.108	ns	12 (25.0)	0.25 (0.12–0.52)	*P* *=* *0.0001*	*0.004*
158V/VV	1 (5.0)	0.19 (0.02–1.50)	P = 0.115	ns	6 (12.5)	0.41 (0.16–1.07)	P = 0.069	ns
Allele carriage								
≥1 158F allele	19 (95.0)	3.57 (0.46–27.48)	P = 0.222	ns	42 (87.5)	1.31 (0.52–3.31)	P = 0.562	ns
≥1 158V allele	9 (45.0)	0.39 (0.16–0.99)	*P* *=* *0.048*	ns	18 (37.5)	0.29 (0.15–0.55)	*P* *=* *0.0001*	*0.004*
*FcγRIIIb*			P = 0.320	ns			P = 0.123	ns
Genotype								
HNA1a+/1b−/1c−	6 (30.0)	2.82 (0.67–11.82)	P = 0.155	ns	9 (18.8)	0.79 (0.33–1.94)	P = 0.612	ns
HNA1a−/1b+/1c−	1 (5.0)	1.04 (0.10–10.53)	P = 0.971	ns	3 (6.3)	0.59 (0.16–2.20)	P = 0.429	ns
HNA1a−/1b−/1c+	0 (0)	–			0 (0)	–		
HNA1a+/1b+/1c− (ref)	3 (15.0)	1			16 (33.3)	1		
HNA1a+/1b−/1c+	4 (20.0)	2.40 (0.51–11.26)	P = 0.267	ns	6 (12.5)	0.68 (0.24–1.86)	P = 0.448	ns
HNA1a−/1b+/1c+	5 (25.0)	5.45 (1.21–24.66)	*P* *=* *0.028*	ns	12 (25.0)	2.45 (1.01–5.96)	*P* *=* *0.047*	ns
HNA1a+/1b+/1c+	1 (5.0)	2.00 (0.19–20.85)	P = 0.562	ns	2 (4.2)	0.75 (0.15–3.68)	P = 0.723	ns
Allele carriage								
≥1 HNA1a allotype	14 (70.0)	0.79 (0.29–2.14)	P = 0.638	ns	33 (68.8)	0.74 (0.38–1.46)	P = 0.388	ns
≥1 HNA1b allotype	10 (50.0)	0.81 (0.33–2.03)	P = 0.659	ns	33 (68.8)	1.79 (0.92–3.47)	P = 0.085	ns
≥1 HNA1c allotype	10 (50.0)	1.69 (0.68–4.22)	P = 0.262	ns	20 (41.7)	1.21 (0.64–2.27)	P = 0.560	ns

P values less than 0.05 are indicated in italics

*P*_*Bonf*_ Bonferroni corrected P value, *OR* odds ratio, *CI* confidence interval, *ns* not statistically significant, –, the variable of interest was not detected in any of the cases and thus could not be analysed

**Table 3:** Maternal FcγR variants associated with perinatal HIV-1 transmission after adjusting for confounding variables

	Total transmitting				Intrapartum transmitting			
	Univariate	Adjusted for VL^a^		P_Bonf_	Univariate	Adjusted for VL		P_Bonf_
		AOR (95 % CI)	P value			AOR (95 % CI)	P value	
*FcγRIIa (rs1801274)*								
Genotype								
131HH (ref)		1				1		
131HR	P = 0.378	1.81 (0.82–3.99)	P = 0.141	ns	P = 0.558	1.43 (0.46–4.46)	P = 0.539	ns
131RR	P = 0.172	2.59 (1.14–5.87)	*P* *=* *0.023*	ns	P = 0.371	2.57 (0.80–8.26)	P = 0.113	ns
Allele carriage								
≥1 131H allele	P = 0.288	0.58 (0.33–1.05)	P = 0.071	ns	P = 0.464	0.49 (0.21–1.16)	P = 0.106	ns
≥1 131R allele	P = 0.233	2.11 (1.00–4.42)	*P* *=* *0.049*	ns	P = 0.449	1.82 (0.64–5.23)	P = 0.263	ns
*FcγRIIb (rs1050501)*								
Genotype								
232II (ref)		1				1		
232IT	P = 0.450	1.29 (0.71–2.35)	P = 0.408	ns	P = 0.442	1.60 (0.65–3.93)	P = 0.309	ns
232TT	P = 0.075	2.80 (1.11–7.10)	*P* *=* *0.030*	ns	P = 0.242	3.25 (0.87–12.17)	P = 0.080	ns
Allele carriage								
≥1 232I allele	P = 0.103	0.41 (0.17–0.97)	*P* *=* *0.043*	ns	P = 0.239	0.40 (0.12–1.33)	P = 0.133	ns
≥1 232T allele	P = 0.231	1.49 (0.84–2.62)	P = 0.171	ns	P = 0.317	1.81 (0.77–4.28)	P = 0.175	ns
*FcγRIIIa (rs396991)*								
Genotype								
158F/FF/FF (ref)		1				1		
158FV/FFV/FVV	*P* *=* *0.010*	0.51 (0.28–0.92)	*P* *=* *0.026*	ns	P = 0.672	1.09 (0.45–2.64)	P = 0.850	ns
158V/VV	*P* *=* *0.041*	0.30 (0.11–082)	*P* *=* *0.018*	ns	P = 0.266	0.20 (0.02–1.70)	P = 0.141	ns
Allele carriage								
≥1 158F allele	P = 0.217	2.29 (0.89–5.88)	P = 0.084	ns	P = 0.183	5.22 (0.67–40.41)	P = 0.114	ns
≥1 158V allele	*P* *=* *0.004*	0.46 (0.26–0.82)	*P* *=* *0.008*	ns	P = 0.980	0.89 (0.37–2.12)	P = 0.786	ns
*FcγRIIIb*								
Genotype								
HNA1a+/1b−/1c−	P = 0.315	0.47 (0.20–1.10)	P = 0.083	ns	P = 0.276	0.45 (0.12–1.61)	P = 0.218	ns
HNA1a−/1b+/1c−	P = 0.668	0.90 (0.33–2.46)	P = 0.839	ns	P = 0.837	1.31 (0.35–4.87)	P = 0.683	ns
HNA1a−/1b−/1c+	–	–			–	–		
HNA1a+/1b+/1c− (ref)		1				1		
HNA1a+/1b−/1c+	P = 0.448	0.63 (0.26–1.51)	P = 0.300	ns	P = 0.727	0.68 (0.19–2.42)	P = 0.547	ns
HNA1a−/1b+/1c+	P = 0.066	1.37 (0.59–3.19)	P = 0.466	ns	P = 0.502	1.20 (0.35–4.15)	P = 0.777	ns
HNA1a+/1b+/1c+	P = 0.849	0.42 (0.10–1.71)	P = 0.226	ns	P = 0.916	0.42 (0.05–3.72)	P = 0.433	ns
Allele carriage								
≥1 HNA1a allotype	P = 0.369	0.78 (0.43–1.44)	P = 0.433	ns	P = 0.648	0.73 (0.30–1.75)	P = 0.481	ns
≥1 HNA1b allotype	*P* *=* *0.025*	2.11 (1.16–3.85)	*P* *=* *0.014*	ns	P = 0.099	2.18 (0.90–5.33)	P = 0.086	ns
≥1 HNA1c allotype	P = 0.599	0.95 (0.54–1.68)	P = 0.865	ns	P = 0.869	0.88 (0.38–2.04)	P = 0.759	ns

	In utero transmitting				In utero-enriched transmitting			
	Univariate	Adjusted for VL + bwt		P_Bonf_	Univariate	Adjusted for VL		P_Bonf_
		AOR (95 % CI)	P value			AOR (95 % CI)	P value	
*FcγRIIa (rs1801274)*								
Genotype								
131HH (ref)		1				1		
131HR	P = 0.241	5.74 (0.66–49.93)	P = 0.113	ns	P = 0.447	2.28 (0.84–6.17)	P = 0.105	ns
131RR	P = 0.085	11.46 (1.29–101.86)	*P* *=* *0.029*	ns	P = 0.225	2.82 (1.01–7.89)	*P* *=* *0.048*	ns
Allele carriage								
≥1 131H allele	P = 0.143	0.34 (0.12–0.97)	*P* *=* *0.045*	ns	P = 0.383	0.63 (0.32–1.27)	P = 0.200	ns
≥1 131R allele	P = 0.136	7.65 (0.94–62.32)	P = 0.057	ns	P = 0.314	2.50 (0.97–6.40)	P = 0.057	ns
*FcγRIIb (rs1050501)*								
Genotype								
232II (ref)						1		
232IT	P = 0.434	0.67 (0.22–2.06)	P = 0.487	ns	P = 0.678	1.15 (0.56–2.35)	P = 0.707	ns
232TT	P = 0.153	3.38 (0.73–15.61)	P = 0.119	ns	P = 0.121	2.57 (0.85–7.74)	P = 0.094	ns
Allele carriage								
≥1 232I allele	P = 0.072	0.25 (0.06–1.07)	P = 0.062	ns	P = 0.133	0.42 (0.15–1.18)	P = 0.100	ns
≥1 232T allele	P = 0.883	0.93 (0.34–2.54)	P = 0.891	ns	P = 0.403	1.33 (0.67–2.61)	P = 0.412	ns
*FcγRIIIa (rs396991)*								
Genotype								
158F/FF/FF (ref)		1				1		
158FV/FFV/FVV	P = 0.108	0.60 (0.21–1.71)	P = 0.341	ns	*P* *=* *0.0001*	0.29 (0.14–0.63)	*P* *=* *0.002*	ns
158V/VV	P = 0.115	0.19 (0.02–1.68)	P = 0.135	ns	P = 0.069	0.34 (0.11–0.98)	*P* *=* *0.046*	ns
Allele carriage								
≥1 158F allele	P = 0.222	4.01 (0.48–33.16)	P = 0.198	ns	P = 0.562	1.71 (0.61–4.80)	P = 0.305	ns
≥1 158V allele	*P* *=* *0.048*	0.50 (0.18–1.36)	P = 0.174	ns	*P* *=* *0.0001*	0.31 (0.15–0.62)	*P* *=* *0.001*	*0.042*
*FcγRIIIb*								
Genotype								
HNA1a+/1b−/1c−	P = 0.155	1.44 (0.30–6.85)	P = 0.644	ns	P = 0.612	0.45 (0.16–1.24)	P = 0.124	ns
HNA1a−/1b+/1c−	P = 0.971	1.26 (0.12–13.63)	P = 0.851	ns	P = 0.429	0.66 (0.17–2.56)	P = 0.544	ns
HNA1a−/1b−/1c+	–	–			–	–		
HNA1a+/1b+/1c− (ref)		1				1		
HNA1a+/1b−/1c+	P = 0.267	1.88 (0.37–9.46)	P = 0.442	ns	P = 0.448	0.59 (0.20–1.68)	P = 0.321	ns
HNA1a−/1b+/1c+	*P* *=* *0.028*	3.10 (0.60–15.95)	P = 0.177	ns	*P* *=* *0.047*	1.53 (0.58–4.02)	P = 0.388	ns
HNA1a+/1b+/1c+	P = 0.562	1.10 (0.10–12.45)	P = 0.939	ns	P = 0.723	0.44 (0.08–2.28)	P = 0.326	ns
Allele carriage								
≥1 HNA1a allotype	P = 0.638	0.85 (0.28–2.63)	P = 0.783	ns	P = 0.388	0.79 (0.38–1.64)	P = 0.523	ns
≥1 HNA1b allotype	P = 0.659	1.09 (0.39–3.02)	P = 0.868	ns	P = 0.085	2.23 (1.08–4.62)	*P* *=* *0.031*	ns
≥1 HNA1c allotype	P = 0.262	1.51 (0.55–4.14)	P = 0.420	ns	P = 0.560	1.04 (0.53–2.06)	P = 0.904	ns

^a^The multivariate analysis adjusted for demographic and clinical variables that independently associated with transmission. Due to high correlation with viral load, CD4 T cell counts were not included in the multivariate model

P values less than 0.05 are indicated in italics

*P*_*Bonf*_ Bonferroni corrected P value, *AOR* adjusted odds ratio, *CI* confidence interval, *VL* viral load, *bwt* birth weight, *ns* not statistically significant, –, the variable of interest was not detected in any of the cases and thus could not be analysed

Carriage of at least one maternal FcγRIIIa-158V allele (confers enhanced antibody binding affinity) associated with a reduced odds of perinatal HIV-1 transmission (OR 0.47, 95 % CI 0.28–0.79, P = 0.004). When analysed according to mode of transmission, a similar association was observed for the in utero transmitting group (OR 0.39, 95 % CI 0.16–0.99, P = 0.048) and in utero-enriched transmitting group (OR 0.29, 95 % CI 0.15–0.55, P = 0.0001), but not for the intrapartum transmitting group (OR 1.01, 95 % CI 0.45–2.25, P = 0.980). These associations remained significant for the total transmitting group and in utero-enriched group in the multivariate analysis (P = 0.008 and P = 0.001, respectively) and for the in utero-enriched group after adjustment for multiple comparisons (univariate: P_Bonf_ = 0.004; multivariate: P_Bonf_ = 0.042).

Possession of an FcγRIIIb-HNA1b allele (modulates neutrophil function) significantly associated with an increased odds of HIV-1 transmission in both the univariate analysis (OR 1.87, 95 % CI 1.08–3.21, P = 0.025) and multivariate analysis (P = 0.014). A similar association was observed for the FcγRIIIb-HNA1b|1c genotype in the in utero transmitting group (OR 5.45, 95 % CI 1.21–24.66, P = 0.028) and in utero-enriched transmitting group (OR 2.45, 95 % CI 1.01–5.96, P = 0.047). However, these associations were not significant in the multivariate analysis.

The FcγRIIa-H131R and FcγRIIb-I232T variants did not associate with perinatal HIV-1 transmission in the univariate analysis. However, after adjustment for confounding variables, the FcγRIIa-131RR genotype (receptor has reduced affinity for IgG2) and FcγRIIb-232TT genotype (confers reduced inhibitory capacity) associated with increased odds of HIV-1 transmission (Table 3).

### FcγR variants and susceptibility of the recipient/infant

In addition to an association observed in the mother, the infant FcγRIIIb-HNA1a|b|c variant also associated with susceptibility to HIV-1 acquisition in the infant (P = 0.046). In particular, carriage of least one FcγRIIIb-HNA1b allotype significantly associated with increased susceptibility to HIV-1 acquisition in the univariate analysis (OR 1.91, 95 % CI 1.11–3.30, P = 0.020; Table 4) and multivariate analysis (P = 0.019; Table 5). Conversely, homozygosity for the FcγRIIIb-HNA1a allotype associated with reduced odds of HIV-1 acquisition in the total infected group (OR 0.42, 95 % CI 0.18–0.96, P = 0.040) and intrapartum infected group (OR 0.19, 95 % CI 0.04–0.89, P = 0.035). The protective effect of FcγRIIIb-HNA1a homozygosity was also observed when compared to other allotype combinations, however not all comparisons remained significant in the multivariate analysis (Additional file 2: Table S2).

**Table 4:** FcγR genotypes and allele carriage in HIV-1 exposed-uninfected and infected infants

	Exposed-uninfected	Total infected				Intrapartum infected			
	N (%)	N (%)	OR (95 % CI)	P value	P_Bonf_	N (%)	OR (95 % CI)	P value	P_Bonf_
*FcγRIIa (rs1801274)*	Overall association			P = 0.704	ns			P = 0.907	ns
Genotype									
131HH (ref)	47 (20.0)	19 (24.4)	1			7 (22.6)	1		
131HR	116 (49.4)	36 (46.2)	0.77 (0.40–1.47)	P = 0.426	ns	14 (45.2)	0.81 (0.31–2.13)	P = 0.670	ns
131RR	72 (30.6)	23 (29.5)	0.79 (0.39–1.61)	P = 0.516	ns	10 (32.3)	0.93 (0.33–2.62)	P = 0.895	ns
Allele carriage									
≥1 131H allele	163 (69.4)	55 (70.5)	1.06 (0.60–1.85)	P = 0.848	ns	21 (67.7)	0.93 (0.42–2.07)	P = 0.854	ns
≥1 131R allele	188 (80.0)	59 (75.6)	0.76 (0.42–1.43)	P = 0.414	ns	24 (77.4)	0.86 (0.35–2.11)	P = 0.737	ns
*FcγRIIb (rs1050501)*	Overall association			P = 0.278	ns			P = 0.773	ns
Genotype									
232II (ref)	116 (49.4)	33 (42.3)	1			14 (45.2)	1		
232IT	90 (38.3)	30 (38.5)	1.17 (0.67–2.06)	P = 0.583	ns	12 (38.7)	1.10 (0.49–2.51)	P = 0.811	ns
232TT	29 (12.3)	15 (19.2)	1.82 (0.87–3.79)	P = 0.110	ns	5 (16.1)	1.43 (0.48–4.29)	P = 0.525	ns
Allele carriage									
≥1 232I allele	206 (86.8)	63 (78.6)	0.59 (0.30–1.17)	P = 0.132	ns	26 (83.9)	0.73 (0.26–2.06)	P = 0.554	ns
≥1 232T allele	119 (47.2)	45 (55.7)	1.33 (0.79–2.23)	P = 0.280	ns	17 (54.8)	1.18 (0.56–2.51)	P = 0.660	ns
*FcγRIIIa (rs396991)*	Overall association		P = 0.339		ns			P = 0.964	ns
Genotype									
158F/FF/FF (ref)	86 (36.6)	34 (43.6)	1			12 (38.7)	1		
158FV/FFV/FVV	118 (50.2)	38 (48.7)	0.81 (0.47–1.40)	P = 0.456	ns	15 (48.4)	0.91 (0.41–2.04)	P = 0.821	ns
158V/VV	31 (13.2)	6 (7.7)	0.49 (0.19–1.28)	P = 0.145	ns	4 (12.9)	0.92 (0.28–3.08)	P = 0.899	ns
Allele carriage									
≥1 158F allele	194 (82.6)	72 (92.3)	0.75 (0.44–1.26)	P = 0.272	ns	27 (87.1)	0.91 (0.42–1.97)	P = 0.819	ns
≥1 158V allele	149 (63.4)	44 (56.4)	1.82 (0.73–4.55)	P = 0.198	ns	19 (61.3)	1.03(0.34–3.13)	P = 0.964	ns
*FcγRIIIb*	Overall association			*P* *=* *0.046*	ns			*P* *=* *0.023*	ns
Genotype									
HNA1a+/1b−/1c−	58 (24.7)	9 (11.5)	0.42 (0.18–0.96)	*P* *=* *0.040*	ns	2 (6.5)	0.19(0.04–0.89)	*P* *=* *0.035*	ns
HNA1a−/1b+/1c−	25 (10.6)	7 (9.0)	0.76 (0.29–1.95)	P = 0.565	ns	1 (3.2)	0.22 (0.03–1.81)	P = 0.160	ns
HNA1a−/1b−/1c+	14 (6.0)	4 (5.1)	0.77 (0.23–2.55)	P = 0.672	ns	0 (0)	–		
HNA1a+/1b+/1c− (ref)	73 (31.2)	27 (34.6)	1			13 (41.9)	1		
HNA1a+/1b−/1c+	36 (15.3)	11 (14.1)	0.83 (0.37–1.85)	P = 0.643	ns	7 (22.6)	1.09 (0.40–2.97)	P = 0.863	ns
HNA1a−/1b+/1c+	22 (9.4)	13 (16.7)	1.60 (0.71–3.61)	P = 0.260	ns	7 (22.6)	1.79 (0.63–5.03)	P = 0.272	ns
HNA1a+/1b+/1c+	7 (3.0)	7 (9.0)	2.70 (0.87–8.43)	P = 0.086	ns	1 (3.2)	0.80 (0.09–7.07)	P = 0.843	ns
Allele carriage									
≥1 HNA1a allotype	174 (74.0)	54 (69.2)	0.79 (0.45–1.38)	P = 0.408	ns	23 (74.2)	1.01 (0.43–2.37)	P = 0.986	ns
≥1 HNA1b allotype	127 (54.0)	54 (69.2)	1.91 (1.11–3.30)	*P* *=* *0.020*	ns	22 (71.0)	2.08 (0.92–4.70)	P = 0.079	ns
≥1 HNA1c allotype	79 (33.6)	35 (44.9)	1.61 (0.95–2.71)	P = 0.075	ns	15 (48.4)	1.85 (0.87–3.94)	P = 0.110	ns

	In utero infected				In utero-enriched infected			
	N (%)	OR (95 % CI)	P value	P_Bonf_	N (%)	OR (95 % CI)	P value	P_Bonf_
*FcγRIIa (rs1801274)*			P = 0.265	ns			P = 0.693	ns
Genotype								
131HH (ref)	4 (21.1)	1			12 (25.5)	1		
131HR	6 (31.6)	0.61 (0.16–2.25)	P = 0.456	ns	22 (46.8)	0.74 (0.34–1.62)	P = 0.455	ns
131RR	9 (47.4)	1.47 (0.43–5.04)	P = 0.541	ns	13 (27.7)	0.71 (0.30–1.68)	P = 0.433	ns
Allele carriage								
≥1 131H allele	10 (52.6)	0.49 (0.19–1.26)	P = 0.139	ns	34 (72.3)	1.16 (0.58–2.32)	P = 0.685	ns
≥1 131R allele	15 (78.9)	0.94 (0.30–2.96)	P = 0.912	ns	35 (74.5)	0.73 (0.35–1.51)	P = 0.396	ns
*FcγRIIb (rs1050501)*			P = 0.083	ns			P = 0.218	ns
Genotype								
232II (ref)	7 (36.8)	1			19 (40.4)	1		
232IT	6 (31.6)	1.10 (0.36–3.40)	P = 0.862	ns	18 (38.3)	1.22 (0.61–2.46)	P = 0.577	ns
232TT	6 (31.6)	3.43 (1.07–10.98)	*P* *=* *0.038*	ns	10 (21.3)	2.11 (0.88–5.01)	P = 0.092	ns
Allele carriage								
≥1 232I allele	13 (68.4)	0.31 (0.11–0.87)	*P* *=* *0.026*	ns	37 (78.7)	0.52 (0.23–1.16)	P = 0.110	ns
≥1 232T allele	12 (63.2)	1.67 (0.64–4.39)	P = 0.298	ns	28 (59.6)	1.44 (0.76–2.71)	P = 0.264	ns
*FcγRIIIa (rs396991)*			P = 0.711	ns			P = 0.145	ns
Genotype								
158F/FF/FF (ref)	9 (47.4)	1			22 (46.8)	1		
158FV/FFV/FVV	8 (42.1)	0.65 (0.24–1.75)	P = 0.391	ns	23 (48.9)	0.76 (0.40–1.46)	P = 0.410	ns
158V/VV	2 (10.5)	0.62 (0.13–3.01)	P = 0.550	ns	2 (4.3)	0.25 (0.06–1.14)	P = 0.073	ns
Allele carriage								
≥1 158F allele	17 (89.5)	0.64 (0.25–1.64)	P = 0.354	ns	45 (95.7)	0.66 (0.35–1.23)	P = 0.190	ns
≥1 158V allele	10 (52.6)	1.29 (0.28–5.87)	P = 0.740	ns	25 (53.2)	3.42 (0.79–14.81)	P = 0.100	ns
*FcγRIIIb*			P = 0.182	ns			P = 0.079	ns
Genotype								
HNA1a+/1b−/1c−	3 (15.8)	0.76 (0.17–3.29)	P = 0.709	ns	7 (14.9)	0.63 (0.24–1.66)	P = 0.350	ns
HNA1a−/1b+/1c−	1 (5.3)	0.58 (0.07–5.24)	P = 0.631	ns	6 (12.8)	1.25 (0.43–3.61)	P = 0.678	ns
HNA1a−/1b−/1c+	1 (5.3)	1.04 (0.11–9.62)	P = 0.970	ns	4 (8.5)	1.49 (0.43–5.20)	P = 0.532	ns
HNA1a+/1b+/1c− (ref)	5 (26.3)	1			14 (29.8)	1		
HNA1a+/1b−/1c+	2 (10.5)	0.81 (0.15–4.39)	P = 0.808	ns	4 (8.5)	0.58 (0.18–1.89)	P = 0.365	ns
HNA1a−/1b+/1c+	5 (26.3)	3.32 (0.88–12.52)	P = 0.077	ns	6 (12.8)	1.42 (0.49–4.14)	P = 0.518	ns
HNA1a+/1b+/1c+	2 (10.5)	4.17 (0.68–25.59)	P = 0.123	ns	6 (12.8)	4.47 (1.30–15.31)	*P* *=* *0.017*	ns
Allele carriage								
≥1 HNA1a allotype	12 (63.2)	0.60 (0.23–1.60)	P = 0.307	ns	31 (66.0)	0.70 (0.35–1.33)	P = 0.258	ns
≥1 HNA1b allotype	13 (68.4)	1.84 (0.68–5.01)	P = 0.231	ns	32 (68.1)	1.81 (0.93–3.53)	P = 0.079	ns
≥1 HNA1c allotype	10 (52.6)	2.19 (0.86–5.62)	P = 0.101	ns	20 (42.6)	1.46 (0.77–2.77)	P = 0.243	ns

P values less than 0.05 are indicated in italics

*P*_*Bonf*_ Bonferroni corrected P value, *OR* odds ratio, *CI* confidence interval, *ns* not statistically significant, –, the variable of interest was not detected in any of the cases and thus could not be analysed

**Table 5:** Infant FcγR variants associated with perinatal HIV-1 acquisition after adjusting for confounding variables

	Total infected				Intrapartum infected			
	Univariate	Adjusted for VL^a^		P_Bonf_	Univariate	Adjusted for VL		P_Bonf_
		AOR (95 % CI)	P value			AOR (95 % CI)	P value	
*FcγRIIa (rs1801274)*								
Genotype								
131HH (ref)		1				1		
131HR	P = 0.426	0.79 (0.38–1.62)	P = 0.519	ns	P = 0.670	0.80 (0.27–2.32)	P = 0.685	ns
131RR	P = 0.516	0.84 (0.39–1.83)	P = 0.657	ns	P = 0.895	0.97 (0.31–2.97)	P = 0.951	ns
Allele carriage								
≥1 131H allele	P = 0.848	1.01 (0.55–1.85)	P = 0.970	ns	P = 0.854	0.89 (0.37–2.12)	P = 0.792	ns
≥1 131R allele	P = 0.414	0.81 (0.41–1.59)	P = 0.536	ns	P = 0.737	0.87 (0.32–2.32)	P = 0.774	ns
*FcγRIIb (rs1050501)*								
Genotype								
232II (ref)		1				1		
232IT	P = 0.583	1.29 (0.70–2.39)	P = 0.415	ns	P = 0.811	1.40 (0.57–3.44)	P = 0.469	ns
232TT	P = 0.110	1.97 (0.89–4.37)	P = 0.096	ns	P = 0.525	1.82 (0.56–5.90)	P = 0.317	ns
Allele carriage								
≥1 232I allele	P = 0.132	0.57 (0.28–1.20)	P = 0.140	ns	P = 0.554	0.65 (0.22–1.90)	P = 0.429	ns
≥1 232T allele	P = 0.280	1.46 (0.83–2.57)	P = 0.195	ns	P = 0.660	1.50 (0.65–3.47)	P = 0.344	ns
*FcγRIIIa (rs396991)*								
Genotype								
158F/FF/FF (ref)		1				1		
158FV/FFV/FVV	P = 0.456	0.87 (0.49–1.56)	P = 0.647	ns	P = 0.821	1.14 (0.49–2.66)	P = 0.764	ns
158V/VV	P = 0.145	0.28 (0.08–1.00)	P = 0.051	ns	P = 0.899	0.28 (0.03–2.27)	P = 0.232	ns
Allele carriage								
≥1 158F allele	P = 0.272	3.34 (0.96–11.57)	P = 0.058	ns	P = 0.819	3.89 (0.50–30.31)	P = 0.194	ns
≥1 158V allele	P = 0.198	0.75 (0.43–1.31)	P = 0.311	ns	P = 0.964	0.95 (0.42–2.19)	P = 0.910	ns
*FcγRIIIb*								
Genotype								
HNA1a+/1b−/1c−	*P* *=* *0.040*	0.37 (0.15–0.92)	*P* *=* *0.033*	ns	*P* *=* *0.035*	0.20 (0.04–0.96)	*P* *=* *0.044*	ns
HNA1a−/1b+/1c−	P = 0.565	0.69 (0.25–1.86)	P = 0.459	ns	P = 0.160	0.20 (0.03–1.69)	P = 0.139	ns
HNA1a−/1b−/1c+	P = 0.672	0.70 (0.18–2.78)	P = 0.616	ns	–	–		P = 0.970
HNA1a+/1b+/1c− (ref)		1				1		
HNA1a+/1b−/1c+	P = 0.643	0.73 (0.31–1.72)	P = 0.478	ns	P = 0.863	0.97 (0.33–2.79)	P = 0.949	ns
HNA1a−/1b+/1c+	P = 0.260	1.57 (0.64–3.88)	P = 0.326	ns	P = 0.272	1.80 (0.57–5.71)	P = 0.316	ns
HNA1a+/1b+/1c+	P = 0.086	2.36 (0.63–8.75)	P = 0.201	ns	P = 0.843	–	ns	P = 0.123
Allele carriage								
≥1 HNA1a allotype	P = 0.408	0.79 (0.43–1.46)	P = 0.452	ns	P = 0.986	1.01 (0.40–2.56)	P = 0.981	ns
≥1 HNA1b allotype	*P* *=* *0.020*	2.02 (1.12–3.64)	*P* *=* *0.019*	ns	P = 0.079	1.91 (0.81–4.53)	P = 0.140	ns
≥1 HNA1c allotype	P = 0.075	1.52 (0.86–2.69)	P = 0.146	ns	P = 0.110	1.74 (0.77–3.96)	P = 0.185	ns

	In utero infected				In utero-enriched infected			
	Univariate	Adjusted for VL + bwt		P_Bonf_	Univariate	Adjusted for VL		P_Bonf_
		AOR (95 % CI)	P value			AOR (95 % CI)	P value	
*FcγRIIa (rs1801274)*								
Genotype								
131HH (ref)		1				1		
131HR	P = 0.456	0.71 (0.15–3.25)	P = 0.657	ns	P = 0.455	0.75 (0.32–1.79)	P = 0.520	ns
131RR	P = 0.541	1.87 (0.45–7.79)	P = 0.390	ns	P = 0.433	0.77 (0.30–1.96)	P = 0.581	ns
Allele carriage								
≥1 131H allele	P = 0.139	0.42 (0.15–1.21)	P = 0.108	ns	P = 0.685	1.07 (0.51–2.22)	P = 0.858	ns
≥1 131R allele	P = 0.912	1.17 (0.31–4.58)	P = 0.817	ns	P = 0.396	0.76 (0.34–1.70)	P = 0.503	ns
*FcγRIIb (rs1050501)*								
Genotype								
232II (ref)		1				1		
232IT	P = 0.862	0.80 (0.23–2.74)	P = 0.724	ns	P = 0.577	1.18 (0.56–2.50)	P = 0.658	ns
232TT	P = 0.038	3.53 (0.95–13.14)	P = 0.060	ns	P = 0.092	2.02 (079–5.16)	P = 0.144	ns
Allele carriage								
≥1 232I allele	P = 0.026	0.26 (0.08–0.86)	*P* *=* *0.028*	ns	P = 0.110	0.54 (0.23–1.28)	P = 0.160	ns
≥1 232T allele	P = 0.298	1.33 (0.47–3.77)	P = 0.593	ns	P = 0.264	1.38 (0.70–2.74)	P = 0.353	ns
*FcγRIIIa (rs396991)*								
Genotype								
158F/FF/FF (ref)		1				1		
158FV/FFV/FVV	P = 0.391	0.61 (0.20–1.86)	P = 0.385	ns	P = 0.410	0.74 (0.37–1.49)	P = 0.405	ns
158V/VV	P = 0.550	0.85 (0.16–4.42)	P = 0.842	ns	P = 0.073	0.29 (0.06–1.36)	P = 0.117	ns
Allele carriage								
≥1 158F allele	P = 0.354	0.93 (0.19–4.53)	P = 0.931	ns	P = 0.190	2.91 (0.66–12.92)	P = 0.160	ns
≥1 158V allele	P = 0.740	0.66 (0.23–1.85)	P = 0.425	ns	P = 0.100	0.65 (0.33–1.28)	P = 0.215	ns
*FcγRIIIb*								
Genotype								
HNA1a+/1b−/1c−	P = 0.709	0.77 (0.15–3.86)	P = 0.748	ns	P = 0.350	0.53 (0.18–1.52)	P = 0.234	ns
HNA1a−/1b+/1c−	P = 0.631	0.46 (0.04–4.76)	P = 0.513	ns	P = 0.678	1.13 (0.37–3.42)	P = 0.827	ns
HNA1a−/1b−/1c+	P = 0.970	1.48 (0.14–15.83)	P = 0.744	ns	P = 0.532	1.33 (0.32–5.54)	P = 0.695	ns
HNA1a+/1b+/1c− (ref)		1				1		
HNA1a+/1b−/1c+	P = 0.808	0.65 (0.10–4.10)	P = 0.645	ns	P = 0.365	0.50 (0.15–1.67)	P = 0.259	ns
HNA1a−/1b+/1c+	P = 0.077	4.47 (0.84–23.80)	P = 0.080	ns	P = 0.518	1.50 (0.46–4.92)	P = 0.501	ns
HNA1a+/1b+/1c+	P = 0.123	3.35 (0.40–27.73)	P = 0.262	ns	*P* *=* *0.017*	4.44 (1.14–17.40)	*P* *=* *0.032*	ns
Allele carriage								
≥1 HNA1a allotype	P = 0.307	0.58 (0.19–1.76)	P = 0.337	ns	P = 0.258	0.66 (0.32–1.37)	P = 0.265	ns
≥1 HNA1b allotype	P = 0.231	1.82 (0.63–5.32)	P = 0.271	ns	P = 0.079	2.16 (1.05–4.44)	*P* *=* *0.037*	ns
≥1 HNA1c allotype	P = 0.101	2.16 (0.76–6.14)	P = 0.149	ns	P = 0.243	1.42 (0.71–2.81)	P = 0.321	ns

P values less than 0.05 are indicated in italics

*P*_*Bonf*_ Bonferroni corrected P value, *AOR* adjusted odds ratio, *CI* confidence interval, *VL* viral load, *bwt* birth weight, –, the variable of interest was not detected in any of the cases and thus could not be analysed

^a^The multivariate analysis adjusted for demographic and clinical variables that independently associated with transmission. Due to high correlation with viral load, CD4 T cell counts were not included in the multivariate model

### Linkage disequilibrium at the low affinity *FCGR* gene locus

**Fig. 2:**
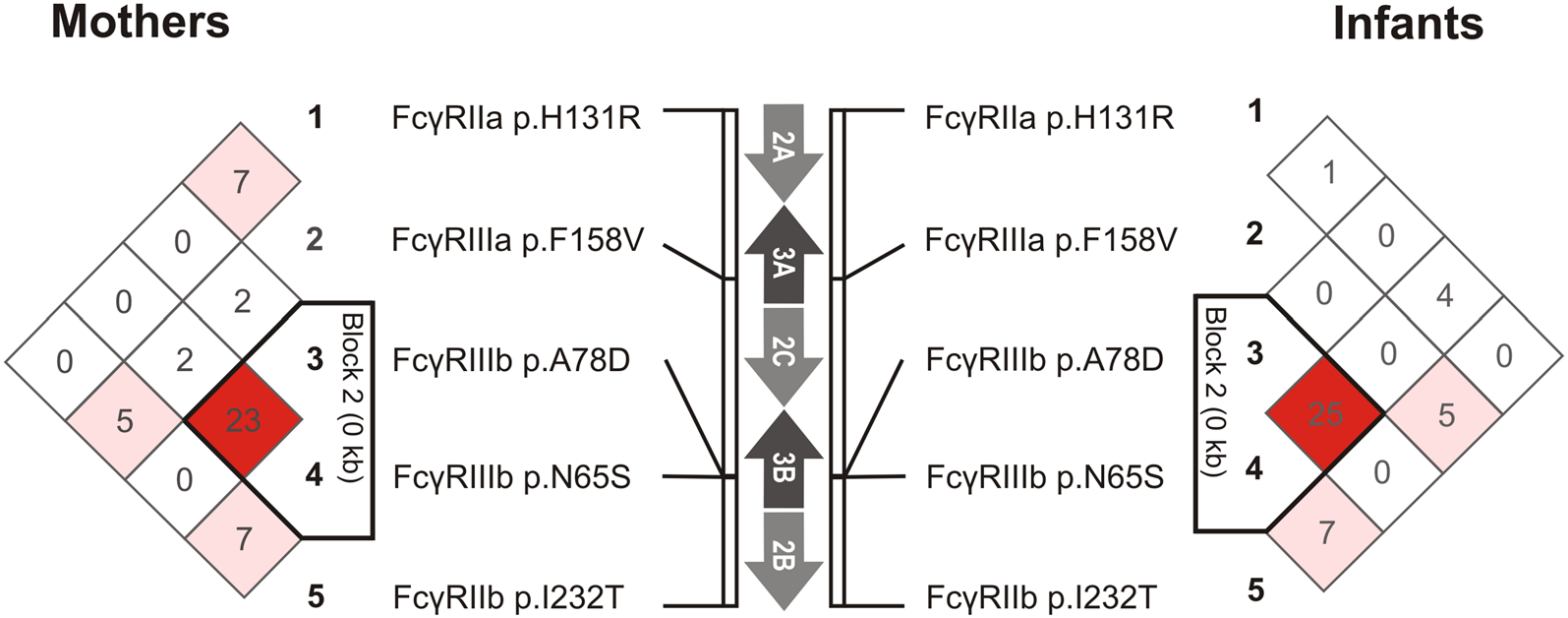
LD for FcγR variants in the study cohort comprising Black South African HIV-1 infected mothers (*left*) and their infants (*right*). Values and colours reflect r^2^ (× 100) and D′/LOD measures of LD, respectively. The *black triangle* depicts a haplotype block that is indicative of the relationship between the FcγRIIIb-HNA1b and -HNA1c allotypes. Such that HNA1b and HNA1c are identical at amino acid position 65 (p.65S) and differ only at amino acid position 78 (p.78A^1b^>D^1c^)

Linkage disequilibrium (LD) between the different FcγR variants could potentially modulate associations observed for the individual FcγRs. Given the strong association of the maternal FcγRIIIa-F158V variant with perinatal HIV-1 transmission, we determined LD in the study cohort (Fig. 2) and adjusted for its possible confounding effect on the associations observed for FcγRIIIb-HNA1a|b|c, FcγRIIa-H131R and FcγRIIb-I232T in the multivariate analysis (Table 6).

**Table 6:** Multivariate analysis adjusted FcγRIIIa-F158V

	Multivariate, not adjusted for FcγRIIIa-F158V	P_Bonf_	Multivariate analysis with adjustment for FcγRIIIa-F158V genotype and allele carriage					
			F158V genotype	P_Bonf_	≥1 158F allele	P_Bonf_	≥1 158V allele	P_Bonf_
***Maternal***								
*FcγRIIa (rs1801274)*								
131RR genotype								
Total transmitting	*P* *=* *0.023*	ns	1.93 (0.82–4.57), P = 0.133	ns	2.25 (0.97–5.24), P = 0.133	ns	2.08 (0.89–4.86), P = 0.091	ns
In utero transmitting	*P* *=* *0.029*	ns	9.37 (1.01–87.22), *P* *=* *0.049*	ns	9.59 (1.05–87.37), *P* *=* *0.045*	ns	10.26 (1.12–94.28), *P* *=* *0.040*	ns
In utero-enriched transmitting	*P* *=* *0.048*	ns	1.94 (0.66–5.70), P = 0.226	ns	2.60 (0.90–7.52), P = 0.077	ns	1.98 (0.67–5.80), P = 0.214	ns
≥1 131H allele								
In utero transmitting	*P* *=* *0.045*	ns	0.42 (0.14–1.29), P = 0.132	ns	0.40 (0.14–1.15), P = 0.088	ns	0.39 (0.13–1.18), P = 0.096	ns
≥1 131R allele								
Total transmitting	*P* *=* *0.049*	ns	1.80 (0.84–3.85), P = 0.128	ns	1.90 (0.89–4.05), P = 0.095	ns	1.91 (0.90–4.06), P = 0.091	ns
*FcγRIIb (rs1050501)*								
232TT genotype								
Total transmitting	*P* *=* *0.030*	ns	2.06 (0.78–5.41), P = 0.144	ns	2.48 (0.96–9.36), P = 0.060	ns	2.17 (0.83–5.67), P = 0.115	ns
≥1 232I allele								
Total transmitting	*P* *=* *0.043*	ns	0.49 (0.20–1.20), P = 0.118	ns	0.43 (0.18–1.05), P = 0.063	ns	0.48 (0.20–1.18), P = 0.110	ns
*FcγRIIIb*								
≥1 HNA1b allotype								
Total transmitting	*P* *=* *0.014*	ns	2.26 (1.22–4.17), *P* *=* *0.009*	ns	2.19 (1.20–4.02), *P* *=* *0.011*	ns	2.21 (1.20–4.11), *P* *=* *0.011*	ns
In utero-enriched transmitting	*P* *=* *0.031*	ns	2.43 (1.15–5.16), *P* *=* *0.020*	ns	2.32 (1.11–4.82), *P* *=* *0.025*	ns	2.40 (1.13–5.10), *P* *=* *0.023*	ns
***Infant***								
*FcγRIIIb*								
HNA1a+/1b−/1c− genotype								
Total infected	*P* *=* *0.033*	ns	0.37 (0.15–0.93), *P* *=* *0.034*	ns	0.37 (0.15–0.91), *P* *=* *0.031*	ns	0.37 (0.15–0.93), *P* *=* *0.034*	ns
Intrapartum infected	*P* *=* *0.044*	ns	0.20 (0.04–0.96), *P* *=* *0.044*	ns	0.19 (0.04–0.95), *P* *=* *0.043*	ns	0.20 (0.04–0.96), *P* *=* *0.044*	ns
HNA1a+/1b+/1c+ genotype								
In utero-enriched infected	*P* *=* *0.032*	ns	5.67 (1.39–23.11), *P* *=* *0.016*	ns	4.47 (1.13–17.64), *P* *=* *0.032*	ns	5.74 (1.39–23.57), *P* *=* *0.015*	ns
≥1 HNA1b allotype								
Total infected	*P* *=* *0.019*	ns	2.11 (1.16–3.83), *P* *=* *0.014*	ns	2.04 (1.12–3.69), *P* *=* *0.019*	ns	2.08 (1.15–3.77), *P* *=* *0.016*	ns
In utero-enriched infected	*P* *=* *0.037*	ns	2.29 (1.10–4.76), *P* *=* *0.026*	ns	2.22 (1.07–4.58), *P* *=* *0.032*	ns	2.26 (1.09–4.68), *P* *=* *0.028*	ns

P values less than 0.05 are indicated in italics

*P*_*Bonf*_ Bonferroni corrected P value, *AOR* adjusted odds ratio, *CI* confidence interval, *VL* viral load, *bwt* birth weight, *ns* not statistically significant

–, the variable of interest was not detected in any of the cases and thus could not be analysed

To determine LD for the FcγRIIIb-HNA1a|b|c allotypes, we used, as a tag-variant, one of four amino acid changes that differentiate HNA1a from HNA1b and HNA1c (p.N^a^65S^bc^, rs448740) as well as the variant that differentiates HNA1c from HNA1a and HNA1b (p.A^ab^78D^c^, rs5030738). The maternal FcγRIIIb-N^a^65S^bc^ variant was not in LD with FcγRIIIa-F158V (P = 0.057, D′ = 0.189, r^2^ = 0.020), while the p.A^ab^78D^c^ variant was in moderate LD with FcγRIIIa-F158V (P = 0.024, D′ = 0.471, r^2^ = 0.029) with the FcγRIIIa-158V allele overrepresented in individuals bearing an FcγRIIIb-78A allele (HNA1c individuals) compared to FcγRIIIb-78DD individuals (59 vs. 20 %). Following adjustment for FcγRIIIa-F158V in the multivariate analysis, the associations previously observed for the FcγRIIIb-HNA1b allotype strengthened for both the total and in utero-enriched transmitting groups (Table 6). Similarly, significance was retained in the infants with associations strengthening for the FcγRIIIb-HNA1a+|1b+|1c+ genotype in the in utero-enriched infected group and carriage of an HNA1b allotype in the total infected and in utero-enriched infected groups (Table 6). Overall, this suggests that the observed associations between the FcγRIIIb-HNA1a|b|c variant and perinatal HIV-1 transmission are not only independent of FcγRIIIa-F158V, but also potentially negatively confounded by FcγRIIIa-F158V.

Both maternal FcγRIIa-H131R and FcγRIIb-I232T was in moderate LD with FcγRIIIa-F158V (P < 0.0001, D′ = 0.351, r^2^ = 0.077 and P = 0.002, D′ = 0.448, r^2^ = 0.052, respectively), with the FcγRIIIa-158V allele overrepresented in individuals bearing an FcγRIIa-131H allele compared to FcγRIIa-131RR individuals (66 vs. 39 %) and in individuals bearing an FcγRIIb-232I allele compared to FcγRIIb-232TT individuals (59 vs. 39 %). When adjusted for FcγRIIIa-F158V in the multivariate analysis, all associations for the FcγRIIa-H131R and FcγRIIb-I232T weakened with the majority losing significance (Table 6). This suggests that the associations observed for FcγRIIa-H131R and FcγRIIb-I232T potentially resulted from LD with FcγRIIIa-F158V.

## Discussion

The extent to which FcγR-mediated effector mechanisms contribute to the risk of HIV-1 transmission and acquisition is currently undefined. Through the study of FcγR functional variants we indirectly demonstrated a role for FcγR-mediated effector functions in modulating perinatal HIV-1 transmission and acquisition. Our findings indicate that the FcγRIIIa-F158V variant that alters antibody binding affinity and functional capacity is associated with infectiousness of an HIV-1 infected mother, while the FcγRIIIb-HNA1a|b|c variant that affects neutrophil effector function is associated with both maternal infectiousness and infant susceptibility.

The significance of FcγR-mediated effector functions in maintaining immune homeostasis is validated by the association of functionally significant FcγR variants with immune disorders [18]. Here we describe an association between the high binding FcγRIIIa allele and reduced maternal infectiousness in perinatal transmission of HIV-1. In particular, carriage of the FcγRIIIa-158V allele by the mother was associated with ~50 % reduction in the odds of HIV-1 transmission. The significant association in the in utero-enriched transmission group, but not in the intrapartum group, suggests that the underlying mechanism may be more pronounced at the maternofoetal interface. FcγRIIIa-bearing leukocytes, including natural killer cells, macrophages and γδ T lymphocytes, are readily recruited to the decidua where they likely contribute to eliminating cell-associated HIV-1 through ADCC [19, 20]. While decidual natural killer cells are primarily FcγRIIIa negative during a healthy pregnancy, they likely upregulate FcγRIIIa expression in the presence of HIV-1 as demonstrated for other perinatally transmitted viruses—human cytomegalovirus and hepatitis C virus [21, 22]. Since cell-associated HIV-1 is thought to be more infectious in utero compared to cell-free virus [23], ADCC-mediated killing of HIV-1 infected cells may contribute to protective immunity at the maternofoetal interface. Of consequence, the FcγRIIIa-F158V variant impacts on ADCC capacity, such that the FcγRIIIa-158V allele exhibits enhanced IgG binding and ADCC capacity compared to the FcγRIIIa-158F allele [7, 24]. The decreased in utero transmission risk associated with the FcγRIIIa-158V allele suggests that the enhanced ADCC capacity conferred by this variant may potentiate elimination of cell-associated HIV-1 and reduce the odds of HIV-1 crossing the placenta through cell–cell interactions. However, the role of ADCC and other potential FcγRIIIa-mediated immune mechanisms—systemic or localized—in perinatal HIV-1 transmission needs to be further elucidated.

In contrast to that observed for the FcγRIIIa-F158V variant, an association between the FcγRIIIb-HNA1a|b|c allotype and perinatal HIV-1 transmission was observed in both the mother and infant. The different FcγRIIIb allotypes arise from multiple amino acid substitutions that do not alter antibody binding affinity, but affect the glycosylation and tertiary structure of the receptor [9, 24–26]. Neutrophils from FcγRIIIb-HNA1a homozygous donors have an enhanced phagocytic and respiratory burst capacity compared to neutrophils from FcγRIIIb-HNA1b homozygous donors [27, 28]. In the present study, homozygosity for the FcγRIIIb-HNA1a allotype in the infant was associated with reduced odds of HIV-1 acquisition compared to other allotype combinations. In both mother and infant, carriage of at least one FcγRIIIb-HNA1b allotype was associated with increased odds of HIV-1 acquisition. Since expression of FcγRIIIb is largely restricted to neutrophils, these findings suggest a potential role for neutrophil-mediated FcγR effector functions in modulating perinatal HIV-1 transmission and acquisition. The underlying mechanism may also involve basophils as FcγRIIIb is detected at low levels on a subset of this cell population, although its function here is unknown.

To date, only the FcγRIIa-H131R variant has been studied in perinatal HIV-1 transmission, with an association reported between the FcγRIIa-131HH genotype and increased infant susceptibility [29]. This association was however not observed in the present study. The contrasting findings are likely attributable to study design. In the Brouwer et al. study, infants were considered perinatally infected if PCR positive at or before 4 months of age where in the present study infant infection status was determined up to 6 weeks of age. The implication thereof is that the number of infants that acquired HIV-1 through breastfeeding is likely higher in the Brouwer et al. study compared to the 12.8 % in the present study. If this is the case, the findings of the Brouwer et al. study may be more representative of an association with HIV-1 transmission through breastfeeding, rather than in utero or intrapartum transmission.

Perinatal HIV-1 transmission is an attractive model in which to study the role of antibodies and their effector functions in HIV-1 protective immunity. This represents a natural situation where the individual at risk is passively immunized with HIV-1-specific antibodies through transplacental transfer of IgG [30, 31]. This model also affords the opportunity to study both members of the transmitting dyad, allowing the assessment of factors contributing to the infectiousness of the transmitter (mother) as well as the susceptibility of the recipient (infant). The findings of this study therefore not only highlight additional immunological factors associated with risk of perinatal HIV-1 transmission, but further support a role for FcγR-mediated effector functions in HIV-1 protective immunity. In particular, findings underscore a potential involvement of neutrophils in protection from HIV-1 transmission and a possible role of FcγR-mediated effector functions in modulating the infectiousness of an HIV-1 infected individual. The significance of these findings in the context of sexual transmission will need to be determined.

There are a number of limitations of the current study and areas that require further investigation. Due to the small sample size and number of comparisons performed it is likely that a number of associations are due to chance. However, since the adjustment for multiple comparisons eliminate type I errors at the cost of type 2 errors, we considered it more important to identify potential factors that may play a role in perinatal HIV-1 transmission rather than dismissing these leads as chance variations brought about by multiple comparisons. Nonetheless, when a Bonferroni correction is applied (α = 0.0012), the association with the maternal FcγRIIIa-F158V variant in the in utero-enriched transmitting group remains significant.

## Conclusions

The maternal and infant immune mechanisms involved in modulating the risk of perinatal HIV-1 transmission and acquisition are complex and multifactorial. Using the approach of studying FcγR genetic variants as proxy for functional capability, this study has revealed the potential importance of FcγR-mediated immune mechanisms that likely involve FcγRIIIa-bearing immune cells and neutrophils. The findings of this study need to be validated in larger cohorts, in particular associations that did not retain significance following adjustment for multiple comparisons. Moreover, understanding the role of IgG Fc-mediated mechanisms requires an appreciation for the collective contribution of multiple components in addition to FcγR genetic variants. These include factors such as the magnitude and specificity of maternal HIV-1 specific antibodies, the efficiency of antibody transfer across the placenta, immune cell phenotypes at the sites of HIV-1 exposure, and the impact of the overall immune environment and state of activation on maternal and infant immune responses.

## Methods

### Study populations

All study participants were Black South African individuals. Ethical clearance was obtained from the University of the Witwatersrand Human Research Ethics Committee and the Institutional Review Board of Columbia University. Written informed consent was obtained from all participants.

### Cohort HIV-1 infection status

Maternal HIV-1 RNA levels were determined using the Roche Amplicor RNA Monitor assay version 1.5 (Roche Diagnostic Systems, Inc., Branchburg, New Jersey, USA). CD4^+^ T cell counts were determined using the FACSCount System from Becton–Dickinson (San Jose, CA, USA). Infant samples were tested for HIV-1 DNA using the Roche Amplicor Monitor version 1.5 qualitative PCR assay (Roche Diagnostic Systems).

### *FCGR* gene copy number variability and nucleotide variant detection

Genomic DNA was extracted from EDTA anticoagulated blood samples using the QIAamp DNA Mini Kit (Qiagen, Dusseldorf, Germany). Functional *FCGR* variants were genotyped using the *FCGR*-specific multiplex ligation-dependent probe amplification (MLPA) assay (MRC Holland, Amsterdam, The Netherlands) according to manufacturer’s instructions [19, 20]. The assay detects the genomic copy number of the *FCGR2C*, *FCGR3A* and *FCGR3B* genes and known functional allelic variants that include FcγRIIa-H131R; FcγRIIb-I232T, FcγRIIIa-F158V, FcγRIIIb-HNA1a|b|c, *FCGR2C* expression variants (p.X57Q and c.798+1A>G), and the *FCGR2B/C* promoter variants (c.-386G>C and c.-120T>A). Genotypes assigned to study participants according to the MLPA assay were confirmed on randomly selected samples with nucleotide sequencing or TaqMan^®^ SNP Genotyping Assays (Thermofisher, Life Technologies, Foster City, USA).

### Computational and statistical analysis

Univariate analyses were used to determine the association between FcγR functional variants and perinatal HIV-1 transmission. Multivariate logistic regression was used to adjust for available confounders that were independently significantly associated with HIV-1 transmission i.e. viral load (all groups) and birth weight (in utero transmitting group) (Table 1). Due to high correlation with viral load, CD4 T^+^ cell count was not included in the multivariate model. The *t* test was used to compare normally distributed continuous variables and the Fisher’s exact test for categorical data. All analyses were performed in STATA version 10.1 (StataCorp LP, College Station, USA) and a P value of less than 0.05 was considered statistically significant. Adjustment for multiple comparisons was performed using the Bonferroni correction, which considered 42 independent tests—mothers and infants, three unrelated clinical subgroups, and seven loci (*FCGR3A* gene copy number, *FCGR3B* gene copy number, FcγRIIa-H131R, FcγRIIb-I232T, FcγRIIIa-F158V, FcγRIIIb-HNA1a|b|c, and overall FcγR variability profiles).

LD between pairs of biallelic loci was tested using an expectation–maximization likelihood-ratio test with 16 000 permutations (significance level <0.05) in Arlequin ver 3.5.2.2 [32]. LD coefficients (D′ and r^2^) were determined in Haploview [33]. Only individuals bearing two copies of each low affinity *FCGR* gene were considered. LD with FcγRIIIb-HNA1a|b|c was assessed using two loci: rs448740 (p.N65S; as tag-variant) that differentiates HNA1a (p.65 N) from HNA1b|c (p.65S) and rs5030738 (p.A78D) that differentiates HNA1a|b (p.78A) from HNA1c (p.78D).

## Additional files

10.1186/s12977-016-0272-y Associations of maternal and infant *FCGR3A* and *FCGR3B* gene copy number with perinatal HIV-1 transmission. Univariate and multivariate analysis of associations of maternal and infant *FCGR3A* and *FCGR3B* gene copy number with perinatal HIV-1 transmission.
10.1186/s12977-016-0272-y Association of the FcγRIIIb-HNA1a homozygous genotype with perinatal HIV-1 acquisition when compared to other combinations of FcγRIIIb-HNA allotypes. Univariate and multivariate analysis of associations of the FcγRIIIb-HNA1a homozygous genotype with perinatal HIV-1 acquisition when compared to other combinations of FcγRIIIb-HNA allotypes.

## Abbreviations

ADCC: antibody-dependent cellular cytotoxicity
ADCP: antibody-dependent cellular phagocytosis
AOR: adjusted odds ratio
CI: confidence interval
CNV: copy number variability
DNA: deoxyribonucleic acid
Fc: fragment, crystallisable
FcγR: Fc gamma receptors
HIV: human immunodeficiency virus
HNA: human neutrophil antigen
IgG: immunoglobulin G
MLPA: multiplex ligation-dependent probe amplification
PCR: polymerase chain reaction
RNA: ribonucleic acid
sdNVP: single dose nevirapine
