# Supplementary material for: Perinatal HIV-1 transmission: Fc gamma receptor variability associates with maternal infectiousness and infant susceptibility
Source: Retrovirology. 2016 Jun 10;13:40. doi: 10.1186/s12977-016-0272-y (PMC4902924; doi:10.1186/s12977-016-0272-y)
Supplement: Supplementary file 1 — 10.1186/s12977-016-0272-y Associations of maternal and infant FCGR3A and FCGR3B gene copy number with perinatal HIV-1 transmission. Univariate and multivariate analysis of associations of maternal and infant FCGR3A and FCGR3B gene copy number with perinatal HIV-1 transmission. [file 12977_2016_272_MOESM1_ESM.docx]

**Table S1.** Associations of maternal and infant *FCGR3A* and *FCGR3B* gene copy number with perinatal HIV-1 transmission

|  | **Total Transmitting/Infected** | | |  | ***Intrapartum* Transmitting/Infected** | | |  | ***In utero* Transmitting/Infected** | | |  | ***In utero*-Enriched Transmitting/Infected** | | |
| --- | --- | --- | --- | --- | --- | --- | --- | --- | --- | --- | --- | --- | --- | --- | --- |
| Reference:  2 gene copies | **Univariate** | **AOR (95% CI), P-value**  Adjusted for VL* | **P_Bonf_** |  | **Univariate** | **AOR (95% CI), P-value**  Adjusted for VL | **P_Bonf_** |  | **Univariate** | **AOR (95% CI), P-value**  Adjusted for VL+bwt | **P_Bonf_** |  | **Univariate** | **AOR (95% CI), P-value**  Adjusted for VL | **P_Bonf_** |
| **Maternal** |  |  |  |  |  |  |  |  |  |  |  |  |  |  |  |
| ***FCGR3A* gene copies** |  |  |  |  |  |  |  |  |  |  |  |  |  |  |  |
| ***Overall association*** | *P=.261* |  | ns |  | *P=.681* |  | ns |  | *P=.131* |  | ns |  | *P=.110* |  | ns |
| 1 copy | P=.142 | 5.60 (0.49-66.29), P=.165 | ns |  | - | - | ns |  | P=.080 | 6.95 (0.41-117.6), P=.179 | ns |  | P=.061 | 9.25 (0.79-108.69), P=.077 | ns |
| 3 copies | P=.741 | 1.26 (0.36-4.37), P=.715 | ns |  | P=.590 | 1.60 (0.32-7.95), P=.568 | ns |  | P=.842 | 0.79 (0.09-7.19), P=.837 | ns |  | P=.986 | 0.94 (0.19-4.62), P=.935 | ns |
|  |  |  |  |  |  |  |  |  |  |  |  |  |  |  |  |
| ***FCGR3B* gene copies** |  |  |  |  |  |  |  |  |  |  |  |  |  |  |  |
| ***Overall association*** | *P=.232* |  | ns |  | *P=.655* |  | ns |  | *P=.461* |  | ns |  | *P=.300* |  | ns |
| ≤ 1 copies | P=.214 | 0.34 (0.08-1.57), P=.169 | ns |  | P=.331 | 0.40 (0.05-3.22), P=.390 | ns |  | - | - |  |  | P=.392 | 0.31 (0.04-2.47), P=.270 | ns |
| ≥ 3 copies | P=.327 | 1.06 (0.55-2.06), P=.864 | ns |  | P=.876 | 0.60 (0.19-1.86), P=.376 | ns |  | P=.763 | 1.27 (0.40-3.97), P=.684 | ns |  | P=.227 | 1.45 (0.68-3.06), P=.332 | ns |
|  |  |  |  |  |  |  |  |  |  |  |  |  |  |  |  |
| **Infant** |  |  |  |  |  |  |  |  |  |  |  |  |  |  |  |
| ***FCGR3A* gene copies** |  |  |  |  |  |  |  |  |  |  |  |  |  |  |  |
| ***Overall association*** | *P=.460* |  | ns |  | *P=1.000* |  | ns |  | *P=.300* |  | ns |  | *P=.171* |  | ns |
| 1 copies | P=.270 | 2.55 (0.34-18.97), P=.362 | ns |  | - | - |  |  | P=.142 | 3.88 (0.33-45-77), P=.281 | ns |  | P=.107 | 4.20 (0.56-31.62), P=.163 | ns |
| 3 copies | P=.732 | 0.73 (0.15-3.65), P=.700 | ns |  | P=.952 | 1.00 (0.12-8.51), P=.999 | ns |  | - | - |  |  | P=.677 | 0.60 (0.07-5.12), P=.644 | ns |
|  |  |  |  |  |  |  |  |  |  |  |  |  |  |  |  |
| ***FCGR3B* gene copies** |  |  |  |  |  |  |  |  |  |  |  |  |  |  |  |
| ***Overall association*** | *P=.134* |  | ns |  | ***P=.029*** |  | ns |  | *P=.247* |  | ns |  | *P=.297* |  | ns |
| ≤ 1 copies | P=.066 | 0.23 (0.05-1.00), P.=051 | ns |  | - | - |  |  | - | - |  |  | P=.528 | 0.43 (0.10-1.97), P=.279 | ns |
| ≥ 3 copies | P=.823 | 0.88 (0.43-1.78), P=.722 | ns |  | P=.210 | 0.15 (0.02-1.15), P=.069 | ns |  | P=.747 | 1.15 (0.34-3.93), P=.826 | ns |  | P=.191 | 1.53 (0.71-3.30), P=.273 | ns |
|  |  |  |  |  |  |  |  |  |  |  |  |  |  |  |  |

* The multivariate analysis adjusted for demographic and clinical variables that independently associated with transmission. Due to high correlation with viral load, CD4 T cell counts were not included in the multivariate model

P-values less than 0.05 are indicated in bold

P_Bonf_ – Bonferroni corrected P-value

AOR – adjusted odds ratio; CI – confidence interval; VL – viral load; bwt – birth weight; (-): The variable of interest was not detected in any of the cases and thus could not be analysed
