# Supplementary material for: Perinatal HIV-1 transmission: Fc gamma receptor variability associates with maternal infectiousness and infant susceptibility
Source: Retrovirology. 2016 Jun 10;13:40. doi: 10.1186/s12977-016-0272-y (PMC4902924; doi:10.1186/s12977-016-0272-y)
Supplement: Supplementary file 2 — 10.1186/s12977-016-0272-y Association of the FcγRIIIb-HNA1a homozygous genotype with perinatal HIV-1 acquisition when compared to other combinations of FcγRIIIb-HNA allotypes. Univariate and multivariate analysis of associations of the FcγRIIIb-HNA1a homozygous genotype with perinatal HIV-1 acquisition when compared to other combinations of FcγRIIIb-HNA allotypes. [file 12977_2016_272_MOESM2_ESM.docx]

**Table S2.** Association of the FcγRIIIb-HNA1a homozygous genotype with perinatal HIV-1 acquisition when compared to other combinations of FcγRIIIb-HNA allotypes

| **Reference:**  **HNA1a+/1b-/1c-** | **Total Infected** | | |  | ***Intrapartum* Infected** | | |  | ***In utero* Infected** | | |  | ***In utero*-Enriched Infected** | | |
| --- | --- | --- | --- | --- | --- | --- | --- | --- | --- | --- | --- | --- | --- | --- | --- |
|  | **Univariate** | **AOR (95% CI), P value**  Adjusted for mVL | **P_Bonf_** |  | **Univariate** | **AOR (95% CI), P value**  Adjusted for mVL | **P_Bonf_** |  | **Univariate** | **AOR (95% CI), P value**  Adjusted for mVL+bwt | **P_Bonf_** |  | **Univariate** | **AOR (95% CI), P value**  Adjusted for mVL | **P_Bonf_** |
| HNA1a-/1b+/1c- | P=.290 | 0.52 (0.15-1.85), P=.313 | ns |  | P=.905 | - | ns |  | P=.827 | 1.67 (0.16-18.16), P=.670 | ns |  | P=.256 | 0.35 (0.09-1.39), P=.135 | ns |
| HNA1a-/1b-/1c+ | P=.363 | 0.40 (0.83-1.95), P=.259 | ns |  | - | - |  |  | P=.787 | 0.52 (0.04-5.97), P=.597 | ns |  | P=.214 | 0.27 (0.05-1.45), P=.127 | ns |
| HNA1a+/1b+/1c- | **P=.040** | 0.37 (0.13-1.02), P=.055 | ns |  | **P=.035** | 0.25 (0.05-1.21), P=.085 | ns |  | P=.709 | 0.77 (0.15-3.86), P=.748 | ns |  | P=.350 | 0.48 (0.14-1.64), P=.242 | ns |
| HNA1a+/1b-/1c+ | P=.173 | 0.47(0.15-1.48), P=.197 | ns |  | **P=.037** | 0.27 (0.06-1.45), P=.126 | ns |  | P=.939 | 1.18 (0.18-7.94), P=.862 | ns |  | P=.901 | 0.78 (0.17-3.48), P=.742 | ns |
| HNA1a-/1b+/1c+ | **P=.008** | 0.24 (0.07-0.77), **P=.016** | ns |  | **P=.008** | 0.16 (0.03-0.97), **P=.046** | ns |  | P=.055 | 0.17 (0.03-0.99), **P=.049** | ns |  | P=.181 | 0.28 (0.07-1.23), P=.092 | ns |
| HNA1a+/1b+/1c+ | **P=.004** | 0.15 (0.03-0.64), **P=.011** | ns |  | P=.270 | - | ns |  | P=.086 | 0.23 (0.03-2.02), P=.184 | ns |  | **P=.004** | 0.10 (0.02-0.47), **P=.004** | ns |
| All other combinations | **P=.017** | 0.36 (0.14-0.91), **P=.030** | ns |  | **P=.037** | 0.32 (0.07-1.45), P=.140 | ns |  | P=.388 | 0.63 (0.17-2.40), P=.497 | ns |  | P=.151 | 0.37 (0.12-1.23), P=.081 | ns |

* The multivariate analysis adjusted for demographic and clinical variables that independently associated with transmission. Due to high correlation with viral load, CD4 T cell counts were not included in the multivariate model

P-values less than 0.05 are indicated in bold

P_Bonf_ – Bonferroni corrected P-value

AOR – adjusted odds ratio; CI – confidence interval; VL – viral load; bwt – birth weight; (-): The variable of interest was not detected in any of the cases and thus could not be analysed
